# Severe and Long-Term Side Effects of Radiotherapy for Nasopharyngeal Cancer: A Case Report

**DOI:** 10.7759/cureus.88993

**Published:** 2025-07-29

**Authors:** Mourad Nafaa, Mamoun Lahlou, Amal Miqdadi, Mostapha Noussair, Belyamani Lahcen

**Affiliations:** 1 Emergency Department, Cheikh Khalifa International University Hospital, Casablanca, MAR; 2 Faculty of Medicine, Mohammed VI University of Health Sciences, Casablanca, MAR; 3 Emergency Department, Mohammed VI University of Health Sciences, Casablanca, MAR; 4 Medicine Department, Cheikh Khalifa International University Hospital, Casablanca, MAR; 5 Emergency Department, Mohammed V Military Training Hospital, Rabat, MAR

**Keywords:** complication of treatment, medical icu, radiotherapy (rt), trismus, ucnt

## Abstract

Radiotherapy is a crucial treatment modality for head and neck cancers, demonstrating significant efficacy in tumor control. However, its application can also lead to challenging complications, particularly due to late effects on healthy tissues. These complications arise from cellular damage, scar fibrosis development, and reduced local blood flow caused by radiation-induced vascular changes. Our patient's experience highlights the diverse range of complications, including trismus, hyposalivation, and visual impairments, commonly associated with radiotherapy in this context. Thus, while radiotherapy is indispensable in cancer management, proactive measures are essential to mitigate its adverse effects and optimize patient outcomes. This case aims to illustrate the possible severe late effects of radiotherapy for nasopharyngeal cancer, emphasizing the need for prolonged surveillance and multidisciplinary management strategies to minimize functional sequelae and improve quality of life in long-term survivors.

## Introduction

The incidence of nasopharyngeal cancers is high in the North African region, specifically in Maghreb countries such as Morocco. Radiotherapy is considered the standard treatment in managing nasopharyngeal cancers [1]. However, it is not without morbidity, and complications can arise due to damage to neighboring structures, especially because it is usually diagnosed at a locally advanced stage, where aggressive treatment is the key to improving survival. The radiation field inevitably covers adjacent bone tissue. Moreover, the radiation dose is usually 65 to 70 Gy, which exceeds the tolerance of surrounding tissues [2]. Late complications, although uncommon, can significantly impact patients' quality of life; their recognition is crucial for timely intervention and management. Despite the widespread use of this treatment, data regarding its long-term complications remain scarce, marking the importance of national oncological surveillance. In this report, we describe a rare case of late-onset, multi-system complications following radiotherapy for nasopharyngeal cancer, illustrating how such toxicities may emerge many years after treatment and severely affect functional outcomes.

## Case presentation

**Figure 1:**
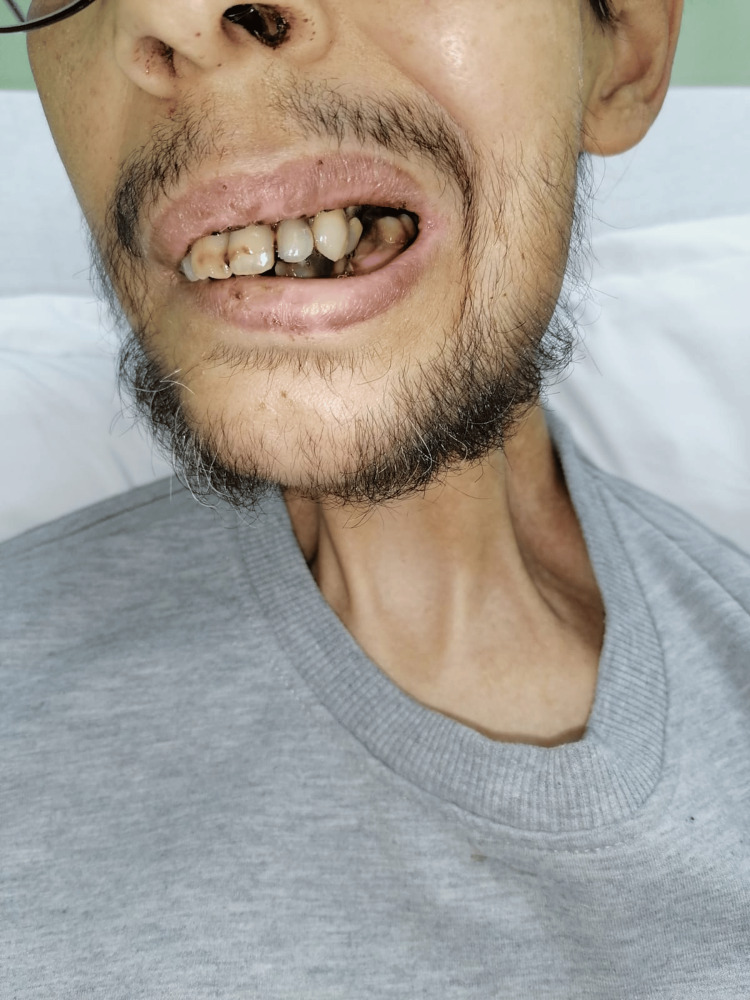
Anterior facial views of the patient.

A 42-year-old male presents with a history of nasopharyngeal carcinoma treated with radiotherapy and chemotherapy in 2004, leading to bilateral hearing impairment, swallowing difficulties with severe hyposalivation grade 3 (Table 1), accompanied by trismus and progressive visual impairments. Since 2018, the patient has been reliant on a silicone nasogastric feeding tube. Symptoms reemerged in July 2023 with a sudden episode of profuse epistaxis due to the removal of the nasogastric tube. The patient initially presented to the emergency department, where hemostasis was achieved by the ENT team through nasal packing, all occurring in a context of deteriorating general health (Figure 1).

**Table 1:** Severity levels and criteria for xerostomia.

Severity level	Criteria
0	No symptoms
1	Symptomatic (thick saliva or dryness without significant alteration in nutrition, unstimulated salivary flow > 0.2 ml/min)
2	Symptomatic with a significant decrease in oral nutrition (excessive water intake, use of lubricant, smooth diet, consumption of water-rich foods), unstimulated salivary flow between 0.1 and 0.2 ml/min
3	Symptoms preventing adequate oral intake, intravenous hydration, nasogastric tube feeding, indication for gastrostomy, unstimulated salivary flow < 0.1 ml/min

In the clinical examination, the patient presented complete trismus, poor dental health, early alopecia, mucositis, bilateral deafness, and cartilaginous naso-buccal mucosa. Nutritional assessment revealed signs of severe malnutrition with a BMI of 17.7 kg/m², likely due to a prolonged enteral feeding and poor oral intake. The patient's general condition was impaired with an ECOG performance status of 3, indicating limited self-care.

**Figure 2:**
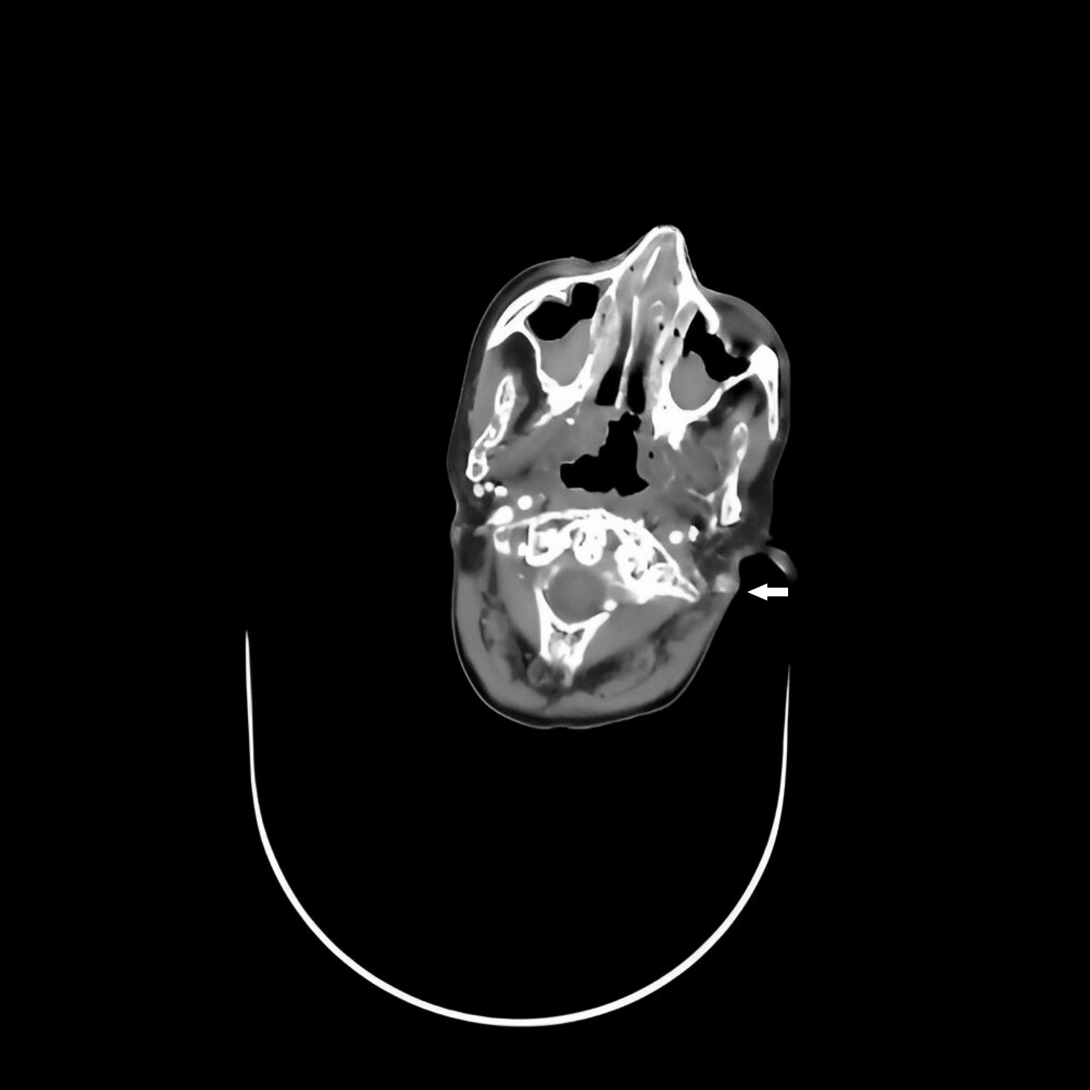
Medical imaging: cervico-facial CT scan revealing the lack of opacification of the left jugular vein and left-sided lateralized cavum parietal rigidity

Cervicofacial CT-scan revealed morphological changes in bones with visualisation of osteonecrosis, left lateralized parietal rigidity of the nasopharynx, probably secondary to radiotherapy local treatment (Figure 2).

**Figure 3:**
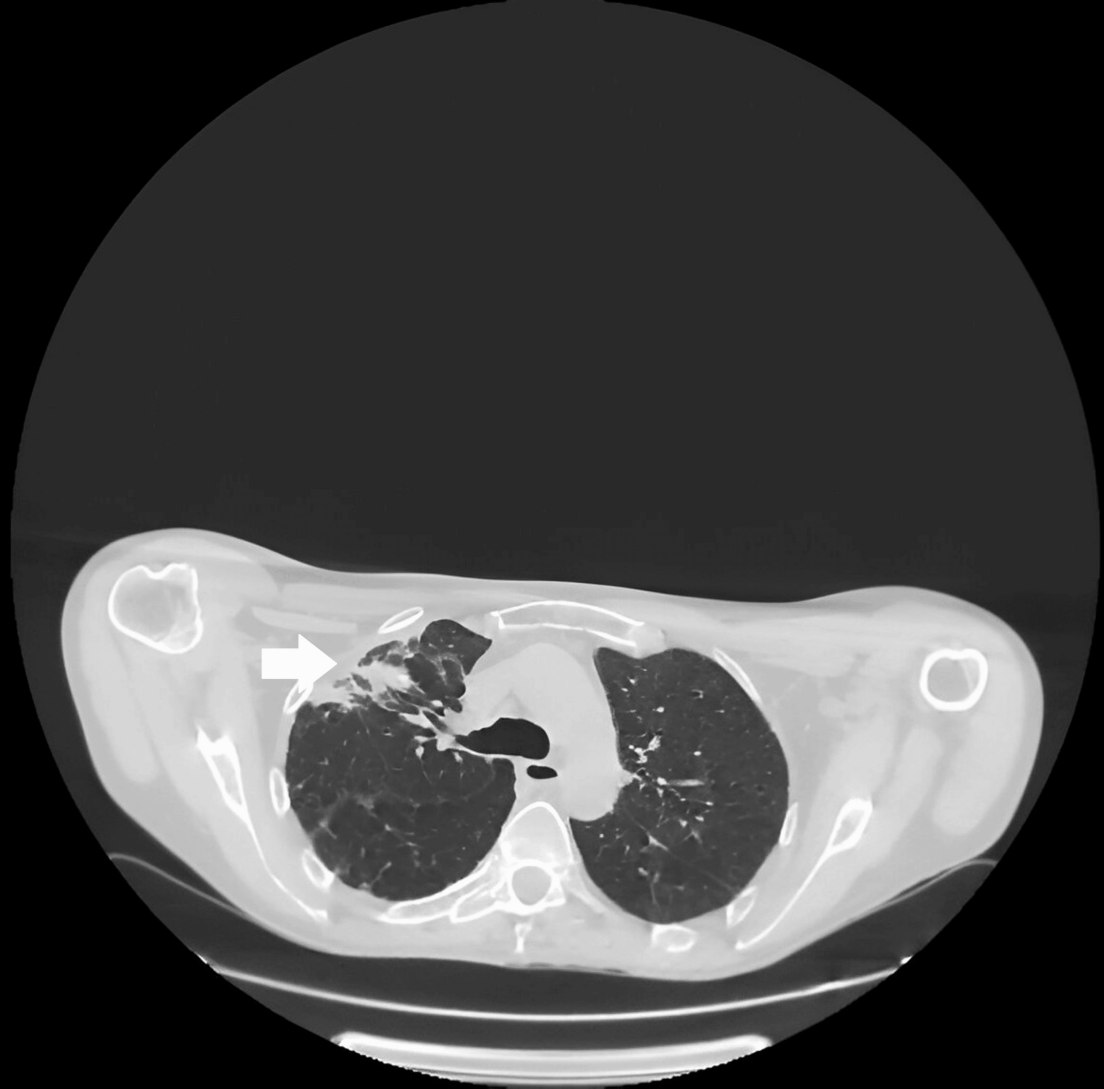
Thoracic CT scans revealing consolidation of the right lung apex with randomly distributed micro-nodules

Thoracic CT-scan showed consolidation of the right pulmonary apex with randomly distributed micro-nodules, and a tissue nodule with smooth contours appears suspicious given the clinical background (likely infectious) (Figure 3).

The patient's laboratory values reveal a hemoglobin level below the normal range (9.6 g/dL), white blood cell count, and platelet count within the normal range (6000/mm³ and 330,000/mm³, respectively). The prothrombin time (PT) and activated partial thromboplastin time (aPTT) are both elevated (74.7 seconds and 1.43 times normal, respectively), indicating potential clotting abnormalities. In addition, the patient exhibited elevated C-reactive protein (CRP) levels (51.70 mg/L) and lower-than-normal levels of sodium, potassium, chloride, and bicarbonate. Urea is below the normal range (0.07 mg/L), while creatinine is slightly elevated (5.50 mg/L). Liver enzyme levels, including aspartate aminotransferase (AST or SGOT) and alanine aminotransferase (ALT or SGPT), are within the normal range (Table 2).

**Table 2:** Laboratory findings

Component	Normal range	Patient value	Status
Hemoglobin (Hb)	13.5-17.5 g/dL	9.6 g/dL	↓ Below normal
White blood cells (WBC)	4.5-11.0 mm³	6000/mm³	→ Within normal
Platelets	150-450 mm³	330,000/mm³	→ Within normal
Prothrombin time (PT)	11-15 seconds	74.7 seconds	↑ Above normal
Activated partial thromboplastin time (aPTT)	25-35 seconds	1.43 times normal	↑ Above normal
Blood type	-	O+	-
C-reactive protein (CRP)	0-5 mg/L	51.70 mg/L	↑ Above normal
Sodium (Na)	135-145 mEq/L	113 mEq/L	↓ Below normal
Potassium (K)	3.5-5.0 mEq/L	2.8 mEq/L	↓ Below normal
Chloride (Cl)	98-108 mEq/L	82 mEq/L	↓ Below normal
Bicarbonate (HCO3)	22-28 mEq/L	20 mEq/L	↓ Below normal
Calcium (Ca)	8.5-10.5 mg/dL	8.4 mg/dL	→ Within normal
Urea	0.15-0.45 mg/L	0.07 mg/L	↓ Below normal
Creatinine (Creat)	5-12 mg/L	5.50 mg/L	→ Within normal
Aspartate aminotransferase (AST or SGOT)	8-40 U/L	26 U/L	→ Within normal
Alanine aminotransferase (ALT or SGPT)	7-56 U/L	11 U/L	→ Within normal

Upon admission to the intensive care unit (ICU), the patient had massive epistaxis, hemodynamic instability, suspected sepsis, and nutritional deterioration. The patient's treatment plan involved several critical interventions. First, posterior nasal packing procedures are initiated by the ENT team to facilitate wound management, bleeding, and infection control. Then, efforts are made to correct any electrolyte imbalances present, aiming to restore normal physiological function. The patient receives a regimen of antibiotics, including ceftriaxone (2 g/24 h), metronidazole (500 mg x 3/24 h), and ciprofloxacin (200 mg x 2/24 h), to treat potential bacterial infections to address the high risk of secondary infections. Furthermore, tranexamic acid (500 mg x 3/24 h) is administered to manage any excessive bleeding tendencies. Continuous clinical and biological monitoring is conducted to assess the patient's condition and response to treatment. Finally, the potential need for a surgical gastrostomy procedure is being evaluated to effectively address the patient's nutritional requirements. Regarding his initial oncological treatment, the patient received radiotherapy and chemotherapy in 2004. At that time, two-dimensional (2D) conventional radiotherapy was the standard approach, as more advanced techniques like 3D conformal radiotherapy or IMRT were not yet in routine use in Morocco. The patient received a total of 70 Gy delivered over 35 fractions (2 Gy per fraction, five days a week), targeting both the primary tumor and the bilateral cervical lymph nodes. Target volumes and dose constraints to organs at risk, such as the parotid glands, cochlea, and retina, were not individually defined, and dosimetric data were not archived.

The patient also received concomitant chemotherapy with cisplatin, following the standard protocol for locally advanced nasopharyngeal carcinoma. It was administered at a dose of 100 mg/m² every three weeks for a total of three cycles during the course of radiotherapy. No neoadjuvant or adjuvant chemotherapy was noted. Due to the time elapsed and the limitations of data storage from that period, further details of treatment planning or in-treatment verification could not be retrieved.

## Discussion

Hyposalivation

At the salivary gland level, irreversible changes occur starting at 26 Gray. Radiation directly affects glandular cells, especially serous parotid cells (apoptotic induction), either through endarteritis or fibrosis. Severe hyposalivation is consistently observed, with partial recovery possible after several years, and depending on the administered dose. It is estimated that >80% of HNC patients exhibit xerostomia and salivary gland hypofunction following RT [3]. Depending on the RT dose, delivery method, and salivary gland-sparing techniques employed, chronic xerostomia affects 64-91% of RT patients with HNC [3,4,5]. There are limited treatment options for RT-induced hyposalivation. The muscarinic receptor agonists pilocarpine and cevimeline that induce saliva secretion from residual acinar cells [6] and artificial saliva provide only temporary symptom relief, which comes at a substantial long-term financial cost [3]. Amifostine is the only FDA-approved radioprotective therapeutic aimed at preventing damage to normal tissues, including salivary glands. However, due to toxicity and potential tumor-protective effects, amifostine is not widely used. The dearth of viable treatment options for averting dysfunction or restoring functionality in irradiated salivary glands is compounded by a constrained understanding of the underlying mechanisms and the multitude of variable responses to diverse radiotherapy (RT) regimens. In this present review, we meticulously explore the intricacies of the mechanisms that contribute to short- and long-term RT-induced dysfunction in salivary glands, concurrently assessing current therapeutic modalities and envisioning auspicious prospects for the future [7].

Trismus

Radiotherapy-induced trismus is one of the most common and severe complications of radiotherapy. The prevalence of trismus after head and neck tumor treatment ranges from 5% to 38%. Radiotherapy induces fibrosis in the masticatory muscles and necrosis of bone and soft tissues, limiting mouth opening. Trismus can precipitate impediments in both feeding and speech, culminating in compromised oral hygiene and notable shifts in facial expression [4]. Furthermore, it can detrimentally influence the nutritional integrity, disrupt oral hydration, and even precipitate aspiration owing to swallowing impairment, thus substantially undercutting the overall quality of life experienced by afflicted patients [4]. In our case, the patient presented with complete trismus, which worsened his nutritional status and oral hygiene, complicating his overall management. In a study by Kraaijenga et al., they studied the relationship between trismus (maximum interincisor opening (MIO) ≤35 mm) and the dose to the ipsilateral masseter muscle (iMM) and ipsilateral medial pterygoid muscle (iMPM) [8]. The results of their study suggested that dose levels of iMM and iMPM were highly correlated due to proximity [8]. Both iMPM and iMM dose parameters were predictive for trismus, especially mean dose and intermediate dose-volume parameters. Optimal cutoffs were 58 Gy (mean dose iMPM), 22 Gy (mean dose iMM), and 46 mm (baseline MIO) [8].

While the exact nature and timing of stimuli implicated in numerous fibrotic diseases remain ambiguous, radiation fibrosis is renowned for its association with both. The initial chemical trigger involves immediate oxidative damage to DNA, proteins, and lipids. Under microscopic scrutiny, fibrosis typically exhibits a focal distribution pattern and is frequently linked to an interstitial fibrin network persisting for many years post-exposure. This prolonged presence is accompanied by evidence of ongoing microvascular injury, emphasizing the chronic nature of the fibrotic process [8]. Observations on the timing of fibrotic changes following radiation exposure in humans are limited by tissue availability (autopsy or surgery); this explains our patient's complete trismus observed on examination, which further worsened his nutritional status and impeded oral care. 

Radio-induced ototoxicity

Ototoxicity, which presents as hearing impairment, tinnitus, and/or vertigo, is a widely acknowledged adverse outcome linked with specific antitumor treatments, notably platinum chemotherapy, radiotherapy, or surgical interventions involving the ear and auditory nerves. Our patient suffers from bilateral deafness that developed progressively after treatment.

Early radio-induced ototoxicity is linked to mucosal edema, inflammation, and desquamation of external, middle, and inner ear tissues [9]. These reactions may manifest acutely or have a delayed onset, potentially affecting all structures within the auditory system and leading to various forms of hearing loss, including conductive, sensorineural, or a combination of both (mixed hearing loss)[9]. During radical irradiation, up to 40% of patients may experience acute middle ear side effects, impacting acoustic structures. Furthermore, approximately one-third of patients may develop late-onset sensorineural hearing loss (SNHL) [9]. Vessels of the inner ear's stria vascularis are affected (arteriocapillary fibrosis and obliterative endarteritis), leading to degeneration, atrophy, and fibrosis of the inner ear's supporting cells. These outer hair cells, located in the first turn of the cochlea (or cochlear base), are fewer and more sensitive to ionizing radiation than inner hair cells. They are responsible for high-frequency hearing, which is more radiosensitive than low-frequency hearing. External radiotherapy in ENT locations leads to ototoxicity in 30-40% of cases. Prevention primarily relies on strict dose constraints at the cochlear level. A dose of 40 Gy should not be exceeded in exclusive radiotherapy cases [5].

Decreased visual acuity

Radio-induced retinopathy is a retinal vessel occlusive disease that appears after irradiation of the eye globe, either for intraocular tumors or neighboring structures' treatment. This condition manifests with a delayed onset (sometimes years) relative to treatment. Microvascular and potentially macrovascular retinal occlusion can lead to decreased visual acuity, retinal neovascularization, and potential secondary enucleation due to painful neovascular glaucoma. According to C. Gilli et al., generally, retinopathies are radio-induced when retinal doses exceed 50 Gy (70 Gy in our context).

Late complications such as radiation-induced retinopathy and glaucoma pose greater severity compared to cataracts, the latter being readily treatable with surgical intervention. Takeda et al. conducted an investigation into the risk of late retinal complications stemming from radiotherapy. Their findings underscored the significance of radiation dosage and the irradiated area in precipitating severe complications. Consequently, it is imperative to minimize radiation dosage (less than 50 Gy) and the area of retinal exposure in patients susceptible to ocular complications [10]. However, following intravitreal bevacizumab (anti-vascular endothelial growth factor treatment), there was observed regression of radiation retinopathy manifestations, including hemorrhage, exudates, intraretinal microangiopathy, and macular edema, without apparent impact on capillary nonperfusion. Notably, a consistent outcome was the reduction in leakage from both preexisting and neovascular retinal vessels, representing a departure from the typical progression of radiation maculopathy [10]. Given the patient's history and imaging findings, his symptomatology likely reflects a cumulative retinal and possibly optic nerve injury due to the radiotherapy.

Mandibular radio-induced osteonecrosis

Since the 1970s, the incidence of mandibular osteoradionecrosis (MORN) associated with radiation therapy has consistently hovered around 3%. MORN typically becomes apparent between two and four years following the completion of radiation treatments [11]. A specific subset of patients may develop persistent, non-healing wounds at the primary tumor site immediately after radiation therapy. These wounds can fail to heal properly and progressively deteriorate, ultimately resulting in conditions such as MORN or chondroradionecrosis [11]. This progression highlights the need for vigilant long-term monitoring and management of patients who have undergone radiation therapy. At the bone level, particularly in the mandible, radiolesions result in decreased osteogenic activity. Osteoporosis is often observed, with cortical thinning and trabecular bone lamellae reduction [12]. Less radiosensitive osteoclasts play a predominant role, forming osteolysis gaps around osteocytes and causing microfractures. Osteoradionecrosis involves progressive bone destruction with mucosal ulceration and exposure. Radiotherapy can additionally cause progressive endarteritis (damaging large- and medium-caliber vessels), as well as hindering the capillary regrowth present in normal healing. This subsequent reduction of tissue perfusion in areas exposed to radiation leads to the protracted open wounds of ORN [12]. The mandible, a flat, poorly vascularized, fragile bone subjected to significant physical stress, is a critically affected organ. According to Piret P et al., complication rates vary between 1% and 44% in various series, averaging below 10% across a compilation of 10,770 cases. However, radionecrosis sometimes occurs spontaneously, without an identified triggering factor, in around 35% of cases.

Perspective and limitations

This case illustrates the long-term consequences of radiotherapy delivered using 2D conventional techniques; advanced methods such as IMRT or VMAT allow better sparing of healthy tissues, greatly reducing these severe complications. However, many patients treated before their adoption remain at risk and require lifelong follow-up and multidisciplinary care. Limitations include a lack of detailed dosimetric data; despite these constraints, the case highlights the need for continued surveillance and documentation of late toxicities in similar contexts.

## Conclusions

In conclusion, radiotherapy stands as a key therapeutic modality, often in combination with chemotherapy, particularly in locally advanced stages, wielding the power to eradicate cancer while protecting the patient's physical integrity through diligent precautionary measures. These enclose strategies such as minimizing radiation exposure duration and implementing protective screens or shields to cover exposed individuals from harm. In our Maghrebian context, nasopharyngeal cancers loom large, and the potential complications of radiotherapy are a cause for considerable concern, given their potential to adversely impact functional prognosis.

This case report illustrates how such complications can manifest many years after initial treatment, underscoring the need to extend the follow-up. It also highlights the importance of early recognition and multidisciplinary management of these sequelae to reduce their impact. However, amidst the complexities of the cervicofacial domain, serious side effects are a rarity, sparing patients from the ominous touch of incapacitating complications. By continuing to advance our understanding, refine preventive strategies, and provide comprehensive care, we can navigate the complexities of radiotherapy with greater efficacy, enhancing outcomes and preserving patients' well-being in the face of formidable challenges.
